# The micro and macro interactions in acute autoimmune encephalitis: a study of resting-state EEG

**DOI:** 10.3389/fneur.2023.1181629

**Published:** 2023-06-08

**Authors:** Xin Luo, Jie Liao, Hong Liu, Qiulin Tang, Hua Luo, Xiu Chen, Jianghai Ruan

**Affiliations:** ^1^Department of Neurology, The Affiliated Hospital of Southwest Medical University, Luzhou, China; ^2^Laboratory of Neurological Diseases and Brain Function, Luzhou, China

**Keywords:** resting-state brain network, acute autoimmune encephalitis, electroencephalography, graph analysis, phase lock value

## Abstract

**Objective:**

Early recognition of autoimmune encephalitis (AIE) is often difficult and time-consuming. Understanding how the micro-level (antibodies) and macro-level (EEG) couple with each other may help rapidly diagnose and appropriately treat AIE. However, limited studies focused on brain oscillations involving micro- and macro-interactions in AIE from a neuro-electrophysiological perspective. Here, we investigated brain network oscillations in AIE using Graph theoretical analysis of resting state EEG.

**Methods:**

AIE Patients (*n* = 67) were enrolled from June 2018 to June 2022. Each participant underwent a ca.2-hour 19-channel EEG examination. Five 10-second resting state EEG epochs with eyes closed were extracted for each participant. The functional networks based on the channels and Graph theory analysis were carried out.

**Results:**

Compared with the HC group, significantly decreased FC across whole brain regions at alpha and beta bands were found in AIE patients. In addition, the local efficiency and clustering coefficient of the delta band was higher in AIE patients than in the HC group (*P* < 0.05). AIE patients had a smaller world index (*P* < 0.05) and higher shortest path length (*P* < 0.001) in the alpha band than those of the control group. Also, the AIE patients' global efficiency, local efficiency, and clustering coefficients decreased in the alpha band (*P* < 0.001). Different types of antibodies (antibodies against ion channels, antibodies against synaptic excitatory receptors, antibodies against synaptic inhibitory receptors, and multiple antibodies positive) showed distinct graph parameters. Moreover, the graph parameters differed in the subgroups by intracranial pressure. Correlation analysis revealed that magnetic resonance imaging abnormalities were related to global efficiency, local efficiency, and clustering coefficients in the theta, alpha, and beta bands, but negatively related to the shortest path length.

**Conclusion:**

These findings add to our understanding of how brain FC and graph parameters change and how the micro- (antibodies) scales interact with the macro- (scalp EEG) scale in acute AIE. The clinical traits and subtypes of AIE may be suggested by graph properties. Further longitudinal cohort studies are needed to explore the associations between these graph parameters and recovery status, and their possible applications in AIE rehabilitation.

## 1. Introduction

Autoimmune encephalitis (AIE), a class of inflammatory encephalopathies, is commonly caused by an immune response against neuronal autoantibodies (1). In addition to behavioral and mental symptoms, autonomic problems, motor disorders, and seizures, AIE can also present with various other clinical presentations (2). Research on AIE has advanced quickly since the discovery of the anti-N-methyl-D-aspartate receptor (anti-NMDAR) antibody (3). A variety of autoantibodies have also been discovered. One of the most frequent forms of encephalitis (AIE) is anti-NMDAR encephalitis, which is followed by anti-Leucine-rich glioma inactivated 1 (anti-LGI-1) and anti-Gamma-amino butyric acid receptor (anti-GABAR) encephalitis. Following the location of the antigen they are targeting, AIEs are commonly divided into three categories: antibodies against synaptic receptors (SyAab), antibodies against intracellular antigens (IncAab), antibodies against ion channels and other cell-surface proteins (CSAab) (4). Targeting molecules involved in neurotransmission that result in dysfunctional neurons is the pathological mechanism that causes AIE. Neurotransmitter release changes are thought to be influenced by SyAab. In contrast, IncAab (anti-HU, anti-Yo, anti-MA) is most likely not directly causative, but rather an epiphenomenon of the T-cell-mediated immune response (5). Since the cause of AIE is not yet known, the majority of diagnostic procedures, including cranial MRI, Scalp electroencephalogram(EEG), and cerebrospinal fluid (CSF) routine, rely on the presence of positive serum autoantibodies or CSF for a definitive diagnosis (6). However, not all hospitals have access to the same antibody tests, which are expensive and time-consuming. As a result, patients may experience delays in diagnosis or develop new symptoms while waiting for results. For a better clinical outcome and fewer neurological recurrences, early immunotherapy and diagnosis are essential (7). To better understand AIE, it may be useful to investigate objective alterations in the electroencephalogram (EEG) network of AIE patients.

The sensitivity of MRI is only 25–50% (8, 9). With common abnormalities showing up as diffuse or focal slow wave changes, EEG has a higher sensitivity than other tests for the diagnosis of AIE. More recently, extreme delta brushing has been discovered to be a relatively specific EEG change in anti-NMDA receptor encephalitis (10) aiding in the diagnosis of AIE. Functional MRI has been used in some prior studies to examine the changes in brain network connectivity in patients with anti-NMDAR encephalitis. Although no significant abnormalities were discovered in their structural MRIs, their fMRIs revealed damage to the hippocampal functional connectivity (FC), medial temporal lobe brain default network (DMN) separation, and frontotemporal lobe contact damage, among other patterns (11). According to functional MRI studies, anti-LGI-1 encephalitis was linked to significant functional network changes. With altered default patterns, large networks with altered functional connections are also present (12). Furthermore, poor temporal resolution fMRI cannot show the sub-second temporal precision needed for the coordination and integration of processing of neural mechanisms between various regions, such as the communication between regulatory neurons and the interchange of regulatory information (13, 14). However, the majority of studies for AIE patients now use fMRI and have small sample sizes; fewer studies have used EEG data from AIE patients for analysis.

EEG's millisecond time scale may be better suited to capturing the rapidly evolving dynamics of brain networks. Electrophysiological methods, allow for investigating oscillatory activity in specific frequency bands (15). A growing number of researchers have investigated cortical functional networks based on graph theory in recent years (16, 17). Graph theory analysis methods have been used to investigate the properties of brain networks in patients suffering from mental disorders (13, 14, 18, 19). However, only a few studies (20, 21) have used graph theory methods to detect resting-state cortical functional networks in NMDAR encephalitis patients. No studies, in particular, have investigated the brain networks of AIE patients using EEG, nor have the networks of patients with AIE encephalitis treated with different antibodies been compared. “Graph theory” is a technique for characterizing complex networks. A graph is a network that is made up of nodes (“vertices”) and connections (“edges”); it can be described by several parameters, including the clustering coefficient (C) and the characteristic path length (L). Phase-locked values (PLV) are commonly used as a phase interaction measure, and PLV is now widely used in brain network connectivity analysis to investigate potential disease abnormalities (22, 23). As a result, we decided to investigate brain network changes in AIE patients using EEG-based graph theory analysis.

Therefore, the purpose of this study is to use PLV construction matrices to select resting-state EEG data from AIE patients for PLV construction of brain network matrices, analyze brain networks, and perform network property analysis. We hypothesized that acute AIE changes brain networks. As a result, our goal was to investigate whether and how EEG brain networks change.

## 2. Material and methods

### 2.1. Participants

AIE patients who were detected between June 2018 and June 2022 were collected in the department of neurology at the affiliated hospital of Southwest Medical University. The inclusion criteria were developed using previously reported AIE diagnostic standards (4). Clinical characteristics and immunoglobulin G (IgG) anti-neuronal antibodies identified in serum and/or cerebrospinal fluid were used to diagnose all cases. The patient's demographic information as well as their clinical, laboratory, and neuroimaging findings were acquired. The cerebrospinal fluid (CSF) autoantibody screening included anti-neuronal surface antigens (NMDAR, LGI1, contactin-associated protein-like 2 (CASPR2), alpha-amino-3-hydroxyl-5-methyl-4-isoxazole-propionate receptor (AMPAR), and -aminobutyric acid receptor (GABABR) antibodies as well as anti-onconeural antibodies (Abs anti-Hu, Yo, Ri, Amphiphysin, CV2, Ma2) For 2 h, we recorded video EEG data. Exclusion criteria include prior history of other severe neuropsychiatric diseases, and considerable EEG data interference. A standard physical examination was also conducted on healthy controls were included based on their age and sex matching and no neurological or mental disorders were reported in the HC group. Finally, this study involved a total of 67 patients with AIE (aged 37.12 ± 17.01 years) and 78 healthy volunteers (aged 35.59 ± 17.42 years) who were age and sex-matched. The mean score of MMSE is 21.93 ± 6.01 in AIE group (Table 1).

**Table 1:** Sex and age of participants in this study.

	**AIE**	**HC**	**χ2/t**	***P***
Sex (male/female) #	29/38	32/46	0.075	0.784
Age (years, mean ± SD) ##	37.12 ± 17.01	35.58 ± 17.42	0.533	0.595
MMSE (score, mean ± SD)	21.93 ± 6.01	\	\	\

# Chi-square test,

## two sample t-test; MMSE, Mini-mental State Examination. AIE, autoimmune encephalitis; HC, healthy control.

This study was conducted following the 2008 Helsinki Declaration. And the study was approved by the Ethics Committee of the Affiliated Hospital of Southwest Medical University (KY2019233). Written informed consent was obtained from all participants.

### 2.2. EEG data acquisition

The video-EEG data were collected in a semi-isolated room. All participants continuously recorded the video-EEG for 2 h using a 19-channel analog recorder (Galileo EB Neuro with a camera). The electrodes (FP1, FP2, F3, F4, F7, F8, T3, T4, T5, T6, C3, C4, P3, P4, O1, O2, Fz, Cz, and Pz) were placed using a quantified ruler according to the International 10-20 system. The impedance of each electrode was kept at less than 10 k*Ω*. The sampling rate is 500 Hz. A time-locked pattern was used to record video and EEG data. The same settings were used to record two-hour video EEG data for age and sex-matched HCs. Following data collection, two experienced clinicians independently checked the entire dataset and wrote the EEG reports.

### 2.3. EEG data preprocessing

The original EEG data was exported in European Data Format (EDF). The removals of muscle and electromyogram artifacts were automated done using an EEGlab plugin-in AAR (http://germangh.com). Then, the EEG was band-pass filtered at 1-30 Hz using a Hamming window FIR filter. After that, the EEG was recomputed to the common average reference. We obtained five 10-second-epochs of EEG in the resting state with eyes closed for each participant. Finally, the preprocessed EEG data was cut out and saved for subsequent analysis. This pipeline was performed using the MATLAB (R2014a, MathWorks, Inc.) toolbox EEGlab (v13.6.5, http://sccn.ucsd.edu).

### 2.4. PLV-based brain network analysis

PLV is the absolute value of the average phase difference between signals, and it can be used to measure the degree of synchronization of EEG signals in a specific frequency band. Graph theory suggests that effective information exchange can occur between signals in a phase-locked relationship.


(1) $$PLV\left(t\right)=\frac{1}{N}\left|\sum_{n=1}^{N}\exp\left(j\left(\Delta \varphi_{n}\left(t\right)\right)\right)\right|$$

Where N represents the total number of sampling points, and Δ*φ*(t) represents the instantaneous difference between signals x and y at time t. PLV has a value ranging from 0 to 1, with 0 indicating that there is no phase synchronization and the two signals are independent with no obvious synchronization. If, on the other hand, the PLV tends to 1, it indicates that the phase coupling between the two signals is stronger and the degree of synchronization is greater. The PLV between each channel was calculated to construct a 19 × 19 PLV network matrix, and the subsequent network property analysis was performed using 19 EEG electrode channels as nodes for each subject. In this study, we constructed the PLV matrices in four frequency bands: delta (1–4 Hz), theta (4–8 Hz), alpha (8–13 Hz), beta band (13–30 Hz).

### 2.5. Graph theory analysis

The 19 EEG channels were utilized in this investigation as nodes for each person in each frequency band. Additionally, graph theory analysis was performed using the previously created 19 by 19 PLV matrix. For describing the topology of the brain network, we used the Small World Index, Clustering Coefficient, Global Efficiency, shortest path length, and Local Efficiency of the PLV network characteristic. The clustering coefficient and local efficiency gauge the brain's capacity for functional differentiation, while characteristic path length and global efficiency assess the network's overall information processing and transfer capacity and degree of network integration. These graph-based indices were calculated using MATLAB functions embedded in the Brain Connectivity Toolbox (http://www.brain-connectivity-toolbox.net).

### 2.6. Sub-group analysis

The mini-mental state examination (MMSE) scale test, with a maximum score of 30 and a score of 27 considered cognitively impaired, was administered to AIE patients during their hospital stay. The AIE group was divided into two groups based on MMSE results: non-cognitive impairment (NCI) (MMSE score ≥27) and cognitive impairment (CI) (MMSE score <27). Due to shallow comas, two individuals were omitted who had not finished the MMSE scale. There were 46 patients in the CI group (aged 38.85 ± 16.87 years) and 19 patients in the NCI group (aged 30.42 ± 14.25 years).

In addition, the groups were divided based on EEG patterns and intracranial pressure (IP) as measured by lumbar puncture. Two qualified doctors examined the scalp EEG (Ruan and Liu). The subgroup with a normal EEG included AIE patients with a regular heartbeat and no epileptiform discharges. Patients with AIE who had an accelerated slow rhythm, epileptiform discharges, or other abnormal forms were included in the abnormal EEG subgroups. Based on the IP of each patient, we divided AIE patients into two groups: normal IP (IP less than or equal to 180 mmH_2_O but larger than 80 mmH_2_O) and high IP (IP larger than 180 mmH_2_O).

Furthermore, several subtypes of AIE patients were classified based on the type of antibodies found in their CSF fluid (antibodies against ion channels, antibodies against synaptic excitatory receptors, antibodies against synaptic inhibitory receptors, and multiple antibodies positive).

### 2.7. Correlation analysis

Spearman's correlation analysis was done between classified variables (cerebrospinal fluid antibodies, serum antibodies, normal EEG and abnormal EEG, normal MRI and abnormal MRI) and Network attribute parameters. Age and sex were employed as covariates to avoid their possible influences on the Network attribute parameters. The significance threshold was at a *P* < 0.05.

### 2.8. Statistical analysis

Demographic data were expressed as mean ± standard deviation (SD) for measurement data, two-sample *t*-test for comparisons between two groups was used. The Chi-square test was used to compare the composition ratio for count data. *P* < 0.05 was considered statistically significant.

For the comparisons of PLV FC and network properties between groups or subgroups, a generalized linear model (GLM) with age and sex as covariates were used to eliminate the possible interferences of age and gender. To compare the PLV FC and network properties between AIE and HC groups or two subgroups, within AIE, two-sample *t-*tests with FDR correction were used. FDR-corrected *P* < 0.05 was thought of as a significant difference. The effect size was calculated using Cohen's *d*. One-way ANOVA tests were conducted for multiple subgroup comparisons divided by subtypes of antibodies. Then, *Post-hoc* Tukey-Kramer tests were performed for pairwise comparisons. Spearman correlation analysis between clinical traits and network attribute parameters after using GLM regressing covariates out was calculated. FDR correction was used for multiple comparisons for all correlations. The statistical significance threshold was set to *P* < 0.05. All the tests were conducted using MATLAB (R2014a, The MathWorks Inc.).

## 3. Results

### 3.1. Antibody detection and clinical information of the included AIE patients

The AIE patient group included 26 anti-NMDAR IgG patients, ten anti-LGI1 patients, four anti-GABAR patients, four anti-Caspr2 patients, four anti-Hu patients, 11 positive multiple antibodies patients, and 12 negative cerebrospinal fluid antibodies patients. Five and two AIE patients with unilateral or bilateral medial temporal lobe hyperintense signals. 15 AIE patients with multi-focal gray matter or white matter abnormal signals. EEG results showed that five AIE patients had epileptic-form discharges, 32 patients had slow alpha rhythm, 12 patients had slow theta-delta rhythm, and the remaining 23 patients had normal EEG patterns (Table 2).

**Table 2:** Clinical information of the included AIE patients^*^.

**AIE patients (*****n =*** **67)**			
**Clinical traits**	**Cases**	**Clinical traits**	**Cases**
**Cerebrospinal fluid Abs**		**EEG pattern**	
Anti-NMDAR Abs	26	Epileptic discharges	5
Anti-GABAR Abs	4	Slowing rhythm	32
Anti-LGI1 Abs	10	Delta rhythm	12
Anti-Hu Abs	4	Normal EEG	23
Multi-Abs	11	**MRI pattern**	
Neg	12	Unilateral mTL hyperintense	5
**IP**		Bilateral mTL hyperintense	2
IP (70–180 mmH_2_O)	33	Multi-focal GM/WM hyperintense	15
IP (>180 mmH2O)	20	Normal MRI	45

* Abs, Antibodies; NMDAR, N-methyl-D-aspartate receptor; LGI1, Leucine-rich glioma inactivated 1; GABAR, Gamma-amino butyric acid receptor; Neg, negative; CSF, Cerebrospinal fluid; mTL, medial temporal lobe; IP, Intracranial pressure.

### 3.2. Comparisons of brain functional connectivity between AIE and HC

**Figure 1:**
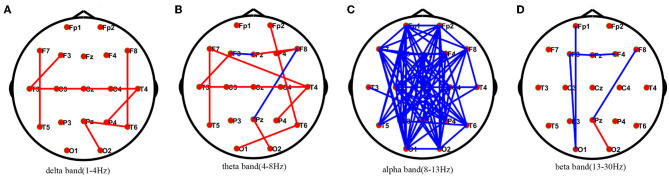
Comparisons of PLV network between AIE and HC patients.* PLV network connectivity in delta(1–4 Hz) **(A)**, theta(4–8 Hz) **(B)**, alpha(8–13 Hz) **(C)**, beta band (13–30 Hz) **(D)**. The red line in the figure indicates the increased connectivity between the temporal regions bilaterally in AIE patients compared to HC in the delta and theta bands. The blue line in the figure indicates that the connections between the left frontal area and the right occipital area and between the right frontal area and the left posterior temporal and occipital areas are reduced in patients with AIE compared to the HC of the alpha band after FDR corrections between the two groups. AIE, autoimmune encephalitis; HC, healthy control.

Compared with healthy individuals, AIE patients had a small number of edges with enhanced connectivity between the left and right temporal regions and between the left and right temporal regions in the delta frequency band; in the theta frequency band, AIE patients had a small number of edges with enhanced connectivity between the bilateral temporal regions; while in the alpha frequency band, AIE patients had a large number of edges with reduced connectivity between the left frontal region and the right occipital region, and between the right frontal region and the left posterior temporal and occipital regions. In the beta band, there are a small number of edges with diminished connectivity between the bilateral frontal and left occipital regions in AIE patients between the two groups (Figure 1).

### 3.3. Comparisons of graph theory parameters between AIE group and HC group

**Figure 2:**
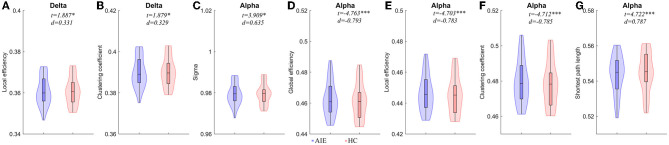
Comparisons of network properties between AIE group and HC group^#^. ^#^**(A, B)** Network Properties of delta band (1–4Hz) between AIE and HC. **(C–G)** Network Properties of alpha band (8–13 Hz) between AIE and HC. Generalized linear models (GLM) with age and sex as covariates were used to assess AIE group and HC group differences. The detailed comparison results of all parameters has been shown in Supplementary Table 1. AIE, autoimmune encephalitis; HC, healthy control; *d*, the effect size Cohen's *d*. *P < 0.05, **P < 0.01, ***P < 0.001. The legends were shown below the figure.

Compared to the HC group, the local efficiency and clustering coefficient of AIE patients increased in the delta band (*P* < 0.05); The small world and shortest path length of alpha band AIE patients were higher than the control group (*P* < 0.05); On the contrary, the parameters of alpha band involving global efficiency, local efficiency, and clustering coefficients in AIE group decreased significantly (*P* < 0.001) (Figure 2, Supplementary Table 1).

Strong Pearson's correlations between these parameters were obtained in both AIE and HC (Supplementary Figures 1 and 2), which may suggest that these Graph parameters detect an important aspect of brain activity. These strong correlations could not be obtained in stimulated EEG (Supplementary Figure 3).

### 3.4. Correlation analysis between graph properties and clinical traits

MRI abnormalities were found to be positively related to global efficiency, local efficiency, and clustering coefficients in theta, alpha, and beta bands, but negatively related to the characteristic path length, MRI patterns were positively correlated with global efficiency in theta band (*r* = 0.286, *P* < 0.05), alpha band (*r* = 0.323, *P* < 0.01), and beta band (*r* = 0.308, *P* < 0.05). Local efficiency in the theta band (*r* = 0.286, *P* < 0.05), alpha band (*r* = 0.322, *P* < 0.01), and beta band (*r* = 0.295, *P* < 0.05) was positively correlated with the MRI performances. Theta (*r* = 0.289, *P* < 0.05), alpha (*r* = 0.322, *P* < 0.01), and beta (*r* = 0.295, *P* < 0.05) clustering coefficients were positively correlated with MRI abnormalities.

### 3.5. Comparisons of graph theory parameters of sub-groups divided by antibodies

**Figure 3:**
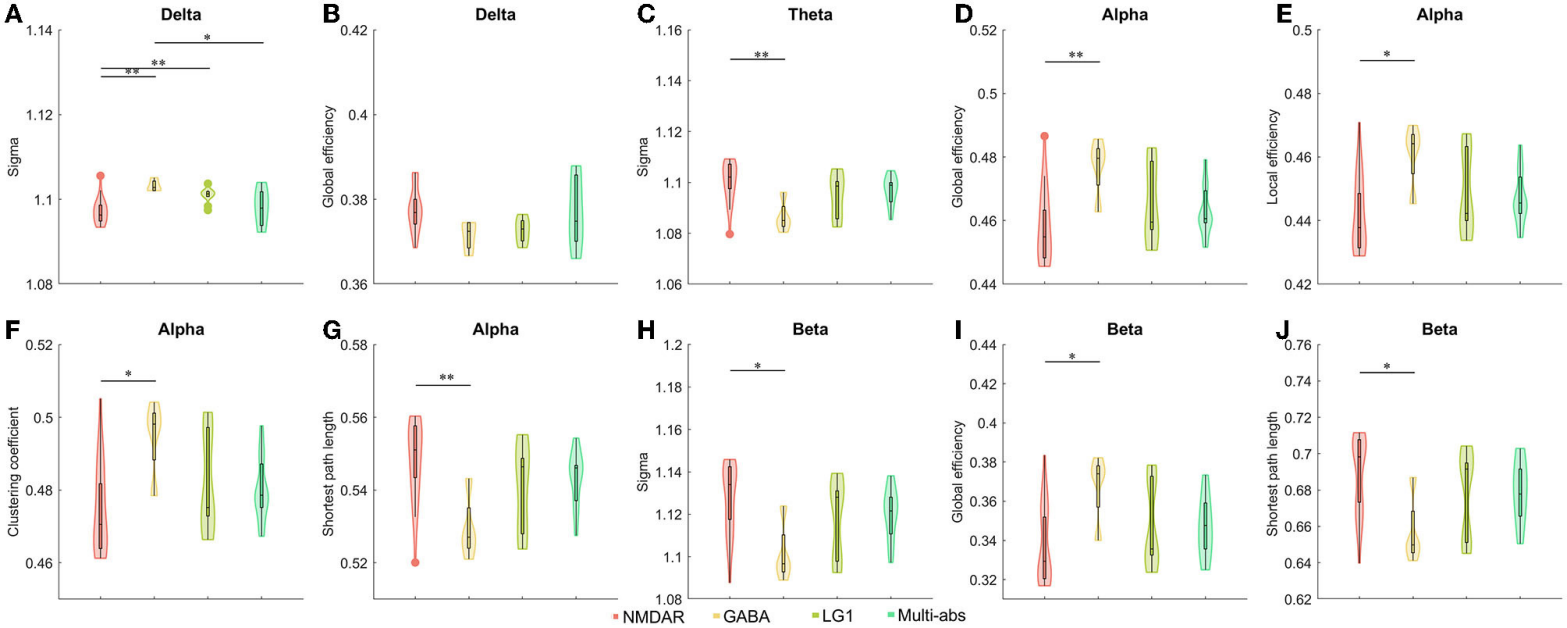
**(A–J)** Comparisons of network properties between subgroups divided by cerebrospinal fluid antibody types in AIE patients^#^. ^#^*post-hoc* Tukey-Kramer tests for pairwise comparisons of sub-groups separated by cerebrospinal fluid antibodies. Antibody types in cerebrospinal fluid such as antibodies against synaptic excitatory receptors, antibodies against synaptic inhibitory receptors, antibodies against ion channels, and multi-antibodies were used to divide AIE patients into sub-groups. As a result, the antibody sub-groups were NMDAR (*n* = 26), GABA (*n* = 4), LG1 (*n* = 10), and Multi-abs (*n* = 11). The line connecting the bars in each panel represented the corresponding pairwise parameter comparison in *post-hoc* Tukey-Kramer tests following one way ANOVA. The results of one-way ANOVA and detailed *post-hoc* tests were shown in Supplementary Tables 2, 3. NMDAR, N-methyl-D-aspartate receptor; GABA, Gamma-amino butyric acid receptor; LGI1, Leucine-rich glioma inactivated 1; Multi-abs, multi-antibody positive; *P < 0.05, **P < 0.01 The legends were shown below the figure.

When we divided the groups by antibodies and antibody characteristics in cerebrospinal fluid, we found statistical differences. We observed the anti-NMDAR AIE showed a significantly decreased small world index in delta band than those of the other three subgroups including anti-GABAR, anti-ion channel and anti-multi-antibody positive AIE (*P* < 0.05). However, compared to anti-GABAR AIE, significantly raised small-world index of theta band (*P* < 0.01) and beta band (*P* < 0.05) in anti-NMDAR AIE group (Figure 3, Supplementary Tables 2, 3).

In the alpha frequency band, there were statistically significant differences in the global efficiency (*P* < 0.01), local efficiency (*P* < 0.05), clustering coefficient (*P* < 0.05), and characteristic path length (*P* < 0.01) of anti-NMDAR encephalitis and anti-GABAR encephalitis. On the beta band, there was a difference in global efficiency and shortest path length between Anti-NMDAR encephalitis and anti-GABAR encephalitis (*P* < 0.05) (Figure 3, Supplementary Tables 2, 3). On the delta band, there was no statistically significant difference in global efficiency between Anti-NMDAR, anti-GABAR, anti-ion channel, and multi-antibody positive AIE.

### 3.6. Comparisons of graph theory parameters of sub-groups divided by IP, EEG, MRI or CI

**Figure 4:**
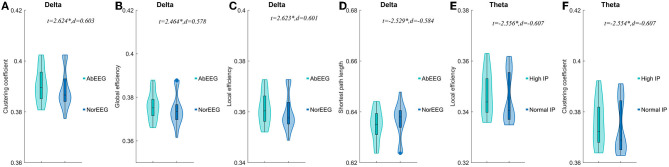
Comparisons of network properties of sub-groups divided by IP and EEG pattern^#^. ^#^Comparison of network properties in the delta band (1–4 Hz) **(A–D)** and theta band (4–8 Hz) (EF). **(A–D)** The AIE sub-groups divided by EEG pattern showed significant differences in network properties of the delta band. **(E, F)**.The AIE sub-groups divided by intracranial pressure showed significant differences in network properties of the theta band. AbEEG, abnormal EEG pattern; NorEEG, normal EEG pattern; IP, intracranial pressure; *d*, the effect size Cohen's *d*. *P < 0.05.

When AIE patients were classified according to clinical EEG reports, AIE patients with abnormal EEG patterns had higher clustering coefficients(*P* < 0.05, *t* = 2.624, *d* = 0.603), global efficiency (*P* < 0.05, *t* = 2.464, *d* = 0.578), local efficiency (*P* < 0.05, *t* = 2.623, *d* = 0.601) on the delta band than AIE patients with normal EEG patterns (*P* < 0.05), while the characteristic path length was instead lower (*P* < 0.05, *t* = –2.529, *d* = −0.584) (Figure 4, Supplementary Table 4).

In the delta band, compared to AIE patients with normal IP, those AIE patients with high IP (>180 mmH_2_O) had significantly lower local efficiency (*P* < 0.05, *t* = –2.556, *d* = –0.607) and clustering coefficients (*P* < 0.05, *t* = –2.554, d = –0.607) in theta band (Figure 4). Other parameters, we not mentioned, did not differ between these subgroups. No significant differences between AIE patients with abnormal MRI and AIE patients with normal MRI, and between AIE patients with CI and AIE patients without CI depicted by MMSE (Supplementary Table 4).

## 4. Discussion

PLV was utilized for phase synchronization, and graph theory has been extensively used in EEG studies. The study showed enhanced FC in the low-frequency band(delta and theta bands)and weakened FC in the high-frequency band (alpha and beta bands), reducing information transfer. Graph theory analysis showed that AIE patients with different types of antibodies had different information transfer efficiency, with significant differences between anti-NMDAR encephalitis and anti-GABAR encephalitis. Subgroups by IP or EEG pattern differed in terms of neural oscillations. These discoveries deepen our comprehension of AIE from a brain electrophysiological perspective.

Studies on FC have identified AIE-related alterations, in which FC is often poorer in AIE (11, 24). Synchronization decline of brain activity in anti-NMDAR encephalitis (25). By gating sensory processing to shield information held online from sensory interference, alpha band oscillations play a significant role in attention and working memory (26). The decreased alpha band connectivity of frontal and parietal areas in AIE could be due to changes in attention and arousal levels (27). The apparent increase in delta and decrease in alpha in psychosis indicates an inappropriate arousal state, which leads to a decreased ability to attend to relevant information (28, 29). Desynchronization of alpha activity is thought to be a reflection of various changes in thalamocortical and cortical network communication (30). The EEG of patients with AIE often presents as a slow wave (7, 31, 32). In anti-NMDAR encephalitis, a significant increase in δ relative band power and a significant decrease in β relative band power and β absolute band power in the posterior region have been reported (33). A higher peak frequency within the delta range was associated with poorer clinical outcomes in anti-NMDAR encephalitis (34). It is widely known that almost all recorded neurons spontaneously oscillate slowly in the delta range when they are anatomically or functionally disconnected from other cortical regions (35). Therefore, an increase in delta connectivity can indicate that AIE is gradually disconnecting.

The neural activity network and information processing in the brain can be explained from this perspective using network property analysis based on graph theory. In the current investigation, we found that in the alpha band, AIE patients had decreased global and local efficiency, increased shortest path length, and decreased clustering coefficients, which is in good agreement with earlier FDG PET and multimodal MRI Studies (36, 37). Given that the thalamus is assumed to be the source of alpha activity, we proposed that the network topology changes in AIE patients that are specific to the alpha band may be connected to changes in thalamic activity (38, 39). These findings are comparable with those of earlier investigations by Bacchi et al. (40) in those individuals with MRI anomalies also had abnormal thalamic signal changes. Many psychiatric symptoms can be caused by injury to the thalamus, which plays an integrative function in cognition. The thalamic-cortical-limbic network is disrupted when the dorsomedial thalamic nucleus, particularly the right one, is damaged. The thalamus loses control over certain portions of the cortex, causing hyperexcitable cortical networks, and the patient's cognitive function is related to the thalamic volume and thalamocortical connections (41, 42). The increased effectiveness of network information transmission in the delta band may be explained by the hypothesis that dysfunction of thalamic oscillations causes hyper synchronization in the band (43, 44).

The normal functioning of GABABR is essential for cognitive function (45). Nearly all patients with anti-GABABR antibody-associated encephalitis have memory loss and other cognitive domain impairment in the acute phase of the disease (46, 47). Cognitive impairment is one of the main manifestations of anti-NMDAR encephalitis, and more than 90% of patients will have cognitive impairment in the acute or subacute phase of the disease, and some patients can have cognitive impairment as the first symptom (48) anti-LGI1 encephalitis cognitive impairment is more frequent, and almost all patients with anti-LGI l antibody-associated encephalitis have comorbid cognitive impairment in the acute or subacute phase (49, 50). The degree of cognitive impairment in VGKC encephalitis is greater than that of GAD65 (51). A few studies have reported varying degrees of cognitive impairment in anti-NMDAR encephalitis, anti-GABA receptor encephalitis, and anti-LGI1 encephalitis (52). It has been shown that a person's functional brain topological features will have a longer shortest path length when they have a lower cognitive capacity (53). Studies have reported a decrease in the local efficiency of the morphological and structural networks of NMDAR encephalitis, which largely accounts for accounts for the patients' cognitive abnormalities (37). The differences in graph theory parameters largely reflect the differences in the cognitive function of different types of antibodies.

The difference in network transmission efficiency could be explained by NMDA-mediated excitatory transmission vs. GABA-mediated inhibitory transmission. NMDA receptors (NMDARs) are glutamate-gated ion channels (the major excitatory neurotransmitter in the CNS). In patients with anti-NMDAR encephalitis, Huang et al. (20) used graph theory analysis to examine the functional connectivity of FDG PET, and they found that most brain regions had considerably reduced global efficiency (20). Wang et al. (37) used multimodal MRI in conjunction with a graph-based network approach to reduce the local efficiency of both morphological and structural networks in anti-NMDAR encephalitis (37). NMDAR-Abs influence glutamate transmission primarily through reversible NMDAR loss, and antibodies cause receptor potency-dependent reductions via cross-linking, capping, and receptor internalization mechanisms. Anti-NMDA antibodies acting on rat hippocampal neurons have also been shown in electrophysiological experiments to reduce NMDA-mediated postsynaptic currents (54, 55). According to retrospective research, 70%−80% of cases involved patients who were initially treated by psychiatrists, and 80–100% of patients reported having psychiatric symptoms at the time of their first presentation (48, 56). Furthermore, psychiatrists have been particularly interested in the central role of NMDAR hypofunction in the emergence of psychotic symptoms. Lebon et al. simulated psychosis based on anti-NMDAR encephalitis (57). Studies have shown that there is a decrease in global and local efficiency in the brain networks of schizophrenic patients compared to normal individuals, and this trend is more pronounced during cognitive load (58). Thus, Network parameter change may be associated with psychiatric symptoms. However, GABAA autoantibodies selectively reduce GABAA receptor surface density and synaptic localization, whereas ionophobic GABAA receptors regulate the majority of rapidly inhibited synaptic transmission in the brain (59–61). Menke et al. found a decrease in the number and amplitude of spontaneous inhibitory postsynaptic currents (sIPSCs) in the hippocampal CA3 network, which resulted in decreased gabaergic synaptic transmission (62). By directly inhibiting GABAA function, these antibodies cause severe encephalitis, resulting in neurological hyperexcitability (63). GABA's nervous system is hyperexcitable, which improves the effectiveness of network messaging. It is well known that seizures are more closely associated with anti-GABAR than with anti-NMDAR encephalitis (64, 65). Anti-GABAR encephalitis starts with seizures in 84% and is characterized by refractory epilepsy and persistent epilepsy (66). Ponten et al. (67) found by studying EEG data from epileptic patients that epileptic activity alters the functional brain network and that an increase in high clustering coefficients can alert for seizures (67). We speculate that differences in clustering coefficients are associated with different antibody seizures in AIE, and further large-scale experiments are needed to verify this.

Topological parameters of the brain network can reflect the patient's intracranial pressure level to some extent. Excessive intracranial pressure impairs the normal function of the cerebral cortex and subcortical tissues, resulting in a reduction in the overall efficiency of the brain network, as well as a decrease in the clustering coefficient and global efficiency. Previous studies of anti-NMDAR encephalitis with MRI abnormalities found a higher severity of disease and worse cognitive function (68) and NMDAR and VGKC encephalitis were found to have a worse prognosis than those with normal MRI (69, 70). AIE patients with abnormal MRIs may have a more severe disease state, resulting in less efficient network information transmission. Autoantibodies in AIE encephalitis cause functional damage to the nervous system (71) but this damage may not change the morphological structure of the brain. Finke C et al. discovered that anti-NMDAR encephalitis manifested as impaired functional connectivity while having normal results on a standard clinical MRI (72). The brain's activity can be sensitively reflected by an EEG. It's also possible that MRIs in AIE patients are normal because the blood-brain barrier disruption and neuronal disruption are both relatively low, and cell-derived edema and vasogenic edema are both mild. Since the normal and abnormal EEGs in this investigation had different network properties, we expected that the AIE patients' EEG network property metrics would be more sensitive or would show abnormalities before the MRI would alter.

When compared to other types of antibody samples, anti-NMDAR encephalitis made up most of our samples. Further longitudinal studies are required to evaluate network variability in AIE patients because our data is cross-sectional. To uncover proof that such EEG recordings could reveal significant disparities, we merely employed 21 EEG electrodes. The results could vary, though, depending on how many electrodes (nodes) are used. To overcome these constraints and develop a more thorough knowledge of AIE in terms of EEG activity, we will keep accumulating cases for future studies.

## 5. Conclusions

In this study, we proved that the brain FC and Graph parameters in resting-state EEG of AIE changed, which were characterized by increased FC in low frequency band, decreased FC in high frequency band and decreased information transmission. Importantly, the micro- (antibodies) scales interacted with macro- (scalp EEG) scale in acute AIE. And the other subgroups divided by IP or EEG pattern also differed in neural oscillations depicted by Graph theory analysis. These observations add to our understanding of how brain functional connectivity and graph parameters change and how the micro- (antibodies) scales interact with macro- (scalp EEG) scale in acute AIE.

## Data availability statement

The raw data supporting the conclusions of this article will be made available by the authors, without undue reservation.

## Ethics statement

The studies involving human participants were reviewed and approved by the Ethics Committee of the Affiliated Hospital of Southwest Medical University. The patients/participants provided their written informed consent to participate in this study.

## Author contributions

XC and JR: conceptualization, supervision, formal analysis, investigation, and review and editing of the manuscript. HLu: supervision and review and editing of the manuscript. XL and JL: data curation, investigation, and writing the original draft of the manuscript. HLi and QT: data curation, formal analysis, and investigation. All authors contributed to the article and approved the submitted version.
