# Supplementary material for: The micro and macro interactions in acute autoimmune encephalitis: a study of resting-state EEG
Source: Front Neurol. 2023 Jun 8;14:1181629. doi: 10.3389/fneur.2023.1181629 (PMC10285084; doi:10.3389/fneur.2023.1181629)
Supplement: Supplementary file 1 [file Data_Sheet_1.DOCX]

**Supplementary Materials:**

The Micro and Macro Interactions in Acute Autoimmune Encephalitis: A Study of Resting-state EEG

**Methods,**

The stimulated EEG was produced using the function (rand) of Matlab. Finally, 100 samples with five epochs of 10-second-length stimulated EEG were created. And then, the phase lock value (PLV) and Graph analysis were done as the patient and control groups.

To detect the correlations between the extracted Graph parameters, Pearson’s correlations (S.Fig.1 and 2) of the Graph parameters between different frequency bands were calculated. FDR correction was used for multiple comparisons for all correlations.

**Surface Laplacian**

We have tried to do spatial filter transformation using Laplacian methods with CSD toolbox （version1.1,https://psychophysiology.cpmc.columbia.edu/software/csdtoolbox/) for the EEG data and calculate the PLV FC again.

**Results**

Strong correlations between these parameters were obtained in autoimmune encephalitis (AIE) and healthy control (HC) groups (S.Fig.1 and 2). No significant correlations of parameters were found in the group with stimulated EEG (S.Fig.3).

From these results after spatial filter, we found the main findings from surface potential and surface Laplacian showed good agreement. The differences between AIE and HC are obtained in delta and alpha bands using the surface potential method have been also found in the results from surface Laplacian method. In addition, the theta bands also showed differences between AIE and HC in surface Laplacian method. That means the main findings of this study still hold true. Furthermore, we found one study have investigate the effects of spatial filters on PLV, and found that the Laplacian eliminated the useful information in zero-phase PLV for task prediction suggesting that it contains effects of both amplitude and phase (W. Jian, M. Chen, and D.J. McFarland, EEG based zero-phase phase-locking value (PLV) and effects of spatial filtering during actual movement. Brain Research Bulletin 130 (2017) 156-164.). Therefore, we keep this result as a reference.

S.Table 1 Comparisons of network properties between AIE group and HC group^#^.

| Graph theory parameters | AIE  n=67 | HC  n=78 | *t* | *d* | *P* |
| --- | --- | --- | --- | --- | --- |
| **Delta band** |  |  |  |  |  |
| sigma | -0.012±0.107 | 0.010±0.063 | -1.442 | -0.249 | 0.137 |
| global efficiency | 0.008±0.088 | -0.007±0.042 | 1.294 | 0.226 | 0.177 |
| local efficiency | 0.012±0.089 | -0.010±0.039 | 1.888 | 0.331 | 0.048 |
| clustering coefficient | 0.012±0.093 | -0.011±0.041 | 1.879 | 0.329 | 0.049 |
| shortest path length | -0.011±0.093 | 0.009±0.044 | -1.625 | -0.284 | 0.090 |
| **Theta band** |  |  |  |  |  |
| sigma | -0.010±0.117 | 0.008±0.060 | -1.153 | -0.201 | 0.230 |
| global efficiency | 0.004±0.093 | -0.003±0.041 | 0.612 | 0.107 | 0.520 |
| local efficiency | 0.009±0.096 | -0.008±0.039 | 1.359 | 0.239 | 0.153 |
| clustering coefficient | 0.009±0.100 | -0.008±0.041 | 1.345 | 0.237 | 0.157 |
| shortest path length | -0.007±0.099 | 0.006±0.045 | -1.049 | -0.184 | 0.271 |
| **Alpha band** |  |  |  |  |  |
| sigma | 0.055±0.129 | -0.047±0.182 | 3.909 | 0.635 | *P<0.001* |
| global efficiency | -0.042±0.100 | 0.036±0.097 | -4.763 | -0.793 | *P<0.001* |
| local efficiency | -0.043±0.103 | 0.037±0.103 | -4.703 | -0.783 | *P<0.001* |
| clustering coefficient | -0.045±0.108 | 0.039±0.107 | -4.712 | -0.785 | *P<0.001* |
| shortest path length | 0.045±0.107 | -0.039±0.108 | 4.722 | 0.787 | *P<0.001* |
| **Beta band** |  |  |  |  |  |
| sigma | -0.002±0.122 | 0.002±0.067 | -0.250 | -0.043 | 0.795 |
| global efficiency | -0.006±0.107 | 0.005±0.047 | -0.757 | -0.133 | 0.427 |
| local efficiency | -0.002±0.112 | 0.002±0.048 | -0.228 | -0.040 | 0.811 |
| clustering coefficient | -0.002±0.118 | 0.002±0.050 | -0.250 | -0.044 | 0.792 |
| shortest path length | 0.003±0.114 | -0.003±0.053 | 0.421 | 0.074 | 0.659 |

^#^Generalized linear models (GLM) with age and sex as covariates were used to assess AIE group and HC group differences. The degree of freedom (df) is 144 in these comparisons. AIE, autoimmune encephalitis; HC, healthy control; *d*, the effect size Cohen’s *d*; The colored values indicated *P*<0.05 after FDR correction.

S.Table 2 One-way ANOVA tests of Graph theory parameters between subgroups divided by cerebrospinal fluid antibody types

| Graph theory parameters | F | P |
| --- | --- | --- |
| **Delta band** |  |  |
| sigma | 7.063 | 0.001 |
| global efficiency | 3.113 | 0.035 |
| local efficiency | 1.241 | 0.306 |
| clustering coefficient | 1.253 | 0.302 |
| shortest path length | 2.036 | 0.122 |
| **Theta band** |  |  |
| sigma | 4.754 | 0.006 |
| global efficiency | 1.853 | 0.151 |
| local efficiency | 1.820 | 0.157 |
| clustering coefficient | 1.828 | 0.155 |
| shortest path length | 2.245 | 0.096 |
| **Alpha band** |  |  |
| sigma | 2.214 | 0.099 |
| global efficiency | 4.211 | 0.010 |
| local efficiency | 3.691 | 0.018 |
| clustering coefficient | 3.705 | 0.018 |
| shortest path length | 4.374 | 0.009 |
| **Beta band** |  |  |
| sigma | 3.445 | 0.024 |
| global efficiency | 2.970 | 0.041 |
| local efficiency | 2.605 | 0.063 |
| clustering coefficient | 2.617 | 0.062 |
| shortest path length | 2.903 | 0.045 |

^#^The subgroups divided by types of cerebrospinal fluid antibodies such as antibodies against synaptic excitatory receptors, antibodies against synaptic inhibitory receptors, antibodies against ion channels, and multi-antibodies were used to divide AIE patients into sub-groups. As a result, the antibody sub-groups were NMDAR (n=26), GABA (n=4), LG1 (n=10), and Multi-abs (n=11). NMDAR, N-methyl-D-aspartate receptor; GABA, Gamma-amino butyric acid receptor; LGI1, Leucine-rich glioma inactivated 1; Multi-abs, multi-antibody positive; The colored values indicated *P*<0.05 after FDR correction. The degree of freedom (df) is 50 in these ANOVA tests.

S.Table 3 The results of post hoc analysis using Tukey-Kramer after ANOVA tests between the four subgroups divided by CSF antibody types^#^.

| Parameters | NMDAR vs. GABA | | NMDAR vs. LG1 | | NMDAR vs. Multi-abs | | GABA vs.LG1 | | GABA vs. Multi-abs | | LG1 vs. Multi-abs | |
| --- | --- | --- | --- | --- | --- | --- | --- | --- | --- | --- | --- | --- |
|  | *Diff.* | *P* | *Diff.* | *P* | *Diff.* | *P* | *Diff.* | *P* | *Diff.* | *P* | *Diff.* | *P* |
| **Delta** |  |  |  |  |  |  |  |  |  |  |  |  |
| sigma | -0.006 | 0.003 | -0.004 | 0.009 | -0.001 | 0.785 | 0.002 | 0.582 | 0.005 | 0.032 | 0.003 | 0.174 |
| GloE | 0.006 | 0.175 | 0.005 | 0.076 | 0.001 | 0.993 | -0.001 | 0.988 | -0.006 | 0.310 | -0.005 | 0.243 |
| locE^##^ | \ | \ | \ | \ | \ | \ | \ | \ | \ | \ | \ | \ |
| Cluco^##^ | \ | \ | \ | \ | \ | \ | \ | \ | \ | \ | \ | \ |
| ShoPL^##^ | \ | \ | \ | \ | \ | \ | \ | \ | \ | \ | \ | \ |
| **Theta band** |  |  |  |  |  |  |  |  |  |  |  |  |
| sigma | 0.014 | 0.006 | 0.006 | 0.175 | 0.003 | 0.561 | -0.008 | 0.258 | -0.010 | 0.089 | -0.002 | 0.898 |
| GloE^##^ | \ | \ | \ | \ | \ | \ | \ | \ | \ | \ | \ | \ |
| locE^##^ | \ | \ | \ | \ | \ | \ | \ | \ | \ | \ | \ | \ |
| Cluco^##^ | \ | \ | \ | \ | \ | \ | \ | \ | \ | \ | \ | \ |
| ShoPL^##^ | \ | \ | \ | \ | \ | \ | \ | \ | \ | \ | \ | \ |
| **Alpha band** |  |  |  |  |  |  |  |  |  |  |  |  |
| sigma^##^ | \ | \ | \ | \ | \ | \ | \ | \ | \ | \ | \ | \ |
| GloE | -0.019 | 0.009 | -0.007 | 0.248 | -0.005 | 0.581 | 0.011 | 0.276 | 0.014 | 0.122 | 0.003 | 0.944 |
| locE | -0.019 | 0.016 | -0.007 | 0.340 | -0.005 | 0.604 | 0.012 | 0.298 | 0.014 | 0.164 | 0.002 | 0.975 |
| Cluco | -0.020 | 0.015 | -0.008 | 0.338 | -0.005 | 0.603 | 0.012 | 0.297 | 0.015 | 0.163 | 0.002 | 0.974 |
| ShoPL | 0.019 | 0.008 | 0.007 | 0.224 | 0.005 | 0.575 | -0.011 | 0.270 | -0.014 | 0.111 | -0.003 | 0.932 |
| **Beta band** |  |  |  |  |  |  |  |  |  |  |  |  |
| sigma | 0.026 | 0.020 | 0.010 | 0.392 | 0.007 | 0.616 | -0.017 | 0.310 | -0.019 | 0.190 | -0.002 | 0.985 |
| GloE | -0.029 | 0.033 | -0.010 | 0.507 | -0.008 | 0.642 | 0.019 | 0.338 | 0.021 | 0.250 | 0.002 | 0.996 |
| locE^##^ | \ | \ | \ | \ | \ | \ | \ | \ | \ | \ | \ | \ |
| Cluco^##^ | \ | \ | \ | \ | \ | \ | \ | \ | \ | \ | \ | \ |
| ShoPL | 0.032 | 0.036 | 0.011 | 0.525 | 0.009 | 0.646 | -0.021 | 0.343 | -0.023 | 0.260 | -0.002 | 0.997 |

^#^The four subgroups divided by types of cerebrospinal fluid antibodies such as antibodies against synaptic excitatory receptors, antibodies against synaptic inhibitory receptors, antibodies against ion channels, and multi-antibodies were used to divide AIE patients into sub-groups. As a result, the antibody sub-groups were NMDAR (n=26), GABA (n=4), LG1 (n=10), and Multi-abs (n=11). NMDAR, N-methyl-D-aspartate receptor; GABA, Gamma-amino butyric acid receptor; LGI1, Leucine-rich glioma inactivated 1; Multi-abs, multi-antibody positive; The paired-comparisons were performed using post hoc Tukey-Kramer tests. ^##^These corresponding parameters showed no significant differences in the ANOVA tests were not taken into post hoc analysis. CSF, cerebrospinal fluid; GloE, global efficiency; locE, local efficiency; Cluco, clustering coefficient; ShoPL, shortest path length; Diff. difference.

S.Table 4 The comparisons of Graph theory parameters between AIE subgroups divided by IP, MRI pattern, EEG pattern and cognitive function ^#^.

^#^IP, intracranial pressure; CI,cognitive impairment; NCI,non-cognitive impairment; *d*, the effect size Cohen’s *d*. GloE, global efficiency; locE, local efficiency; Cluco, clustering coefficient; ShoPL, shortest path length; The colored values indicated *P*<0.05 after FDR correction.

|  | **High IP vs. Normal IP**  **(*df*=53)** | | | **Abnormal MRI vs. Normal MRI(*df*=43)** | | | **Abnormal EEG vs. Normal EEG(*df*=58)** | | | **CI vs. NCI**  **(*df*=51)** | | |
| --- | --- | --- | --- | --- | --- | --- | --- | --- | --- | --- | --- | --- |
|  | *t* | *P* | *d* | *t* | *P* | *d* | *t* | *P* | *d* | *t* | *P* | *d* |
| **Delta** |  |  |  |  |  |  |  |  |  |  |  |  |
| sigma | 0.422 | 0.708 | 0.106 | 0.502 | 0.624 | 0.123 | -2.458 | 0.065 | -0.517 | -0.608 | 0.546 | -0.183 |
| GloE | -1.280 | 0.264 | -0.319 | -0.408 | 0.690 | -0.100 | 2.464 | 0.040 | 0.578 | 1.328 | 0.190 | 0.399 |
| locE | -1.400 | 0.229 | -0.343 | -0.545 | 0.594 | -0.134 | 2.623 | 0.033 | 0.601 | 1.467 | 0.231 | 0.364 |
| Cluco | -1.397 | 0.230 | -0.342 | -0.539 | 0.598 | -0.133 | 2.624 | 0.033 | 0.603 | 1.468 | 0.230 | 0.365 |
| ShoPL | 1.248 | 0.279 | 0.308 | 0.460 | 0.653 | 0.113 | -2.529 | 0.038 | -0.584 | -1.481 | 0.220 | -0.373 |
| **Theta band** |  |  |  |  |  |  |  |  |  |  |  |  |
| sigma | 1.062 | 0.360 | 0.260 | -0.011 | 0.991 | -0.003 | -1.087 | 0.364 | -0.252 | -0.446 | 0.657 | -0.134 |
| GloE | -2.176 | 0.064 | -0.532 | -0.014 | 0.989 | -0.004 | 1.348 | 0.183 | 0.371 | 0.744 | 0.460 | 0.223 |
| locE | -2.556 | 0.036 | -0.607 | -0.031 | 0.975 | -0.008 | 1.821 | 0.117 | 0.437 | 0.670 | 0.506 | 0.201 |
| Cluco | -2.554 | 0.036 | -0.607 | -0.032 | 0.975 | -0.008 | 1.821 | 0.117 | 0.438 | 0.672 | 0.505 | 0.202 |
| ShoPL | 2.248 | 0.058 | 0.545 | -0.020 | 0.984 | -0.005 | -1.470 | 0.147 | -0.404 | -0.666 | 0.508 | -0.200 |
| **Alpha band** |  |  |  |  |  |  |  |  |  |  |  |  |
| sigma | -0.884 | 0.381 | -0.249 | 0.179 | 0.860 | 0.044 | 0.416 | 0.679 | 0.115 | -0.997 | 0.323 | -0.300 |
| GloE | -0.047 | 0.963 | -0.013 | 0.223 | 0.824 | 0.056 | -0.466 | 0.643 | -0.128 | 0.353 | 0.725 | 0.106 |
| locE | -0.363 | 0.746 | -0.092 | 0.037 | 0.971 | 0.009 | -0.265 | 0.792 | -0.073 | 0.140 | 0.889 | 0.042 |
| Cluco | -0.356 | 0.751 | -0.090 | 0.041 | 0.968 | 0.010 | -0.268 | 0.789 | -0.074 | 0.143 | 0.887 | 0.043 |
| ShoPL | 0.065 | 0.948 | 0.018 | -0.142 | 0.888 | -0.035 | 0.392 | 0.696 | 0.108 | -0.273 | 0.786 | -0.082 |
| **Beta band** |  |  |  |  |  |  |  |  |  |  |  |  |
| sigma | 1.078 | 0.371 | 0.255 | 0.337 | 0.737 | 0.084 | -1.568 | 0.221 | -0.340 | -0.814 | 0.519 | -0.195 |
| GloE | -1.889 | 0.117 | -0.449 | -0.176 | 0.861 | -0.044 | 1.128 | 0.356 | 0.256 | 0.518 | 0.607 | 0.156 |
| locE | -2.340 | 0.060 | -0.541 | -0.242 | 0.809 | -0.061 | 1.620 | 0.200 | 0.357 | 0.452 | 0.713 | 0.111 |
| Cluco | -2.337 | 0.060 | -0.541 | -0.243 | 0.809 | -0.061 | 1.617 | 0.200 | 0.357 | 0.456 | 0.710 | 0.112 |
| ShoPL | 2.057 | 0.092 | 0.483 | 0.207 | 0.836 | 0.052 | -1.398 | 0.260 | -0.313 | -0.571 | 0.640 | -0.141 |


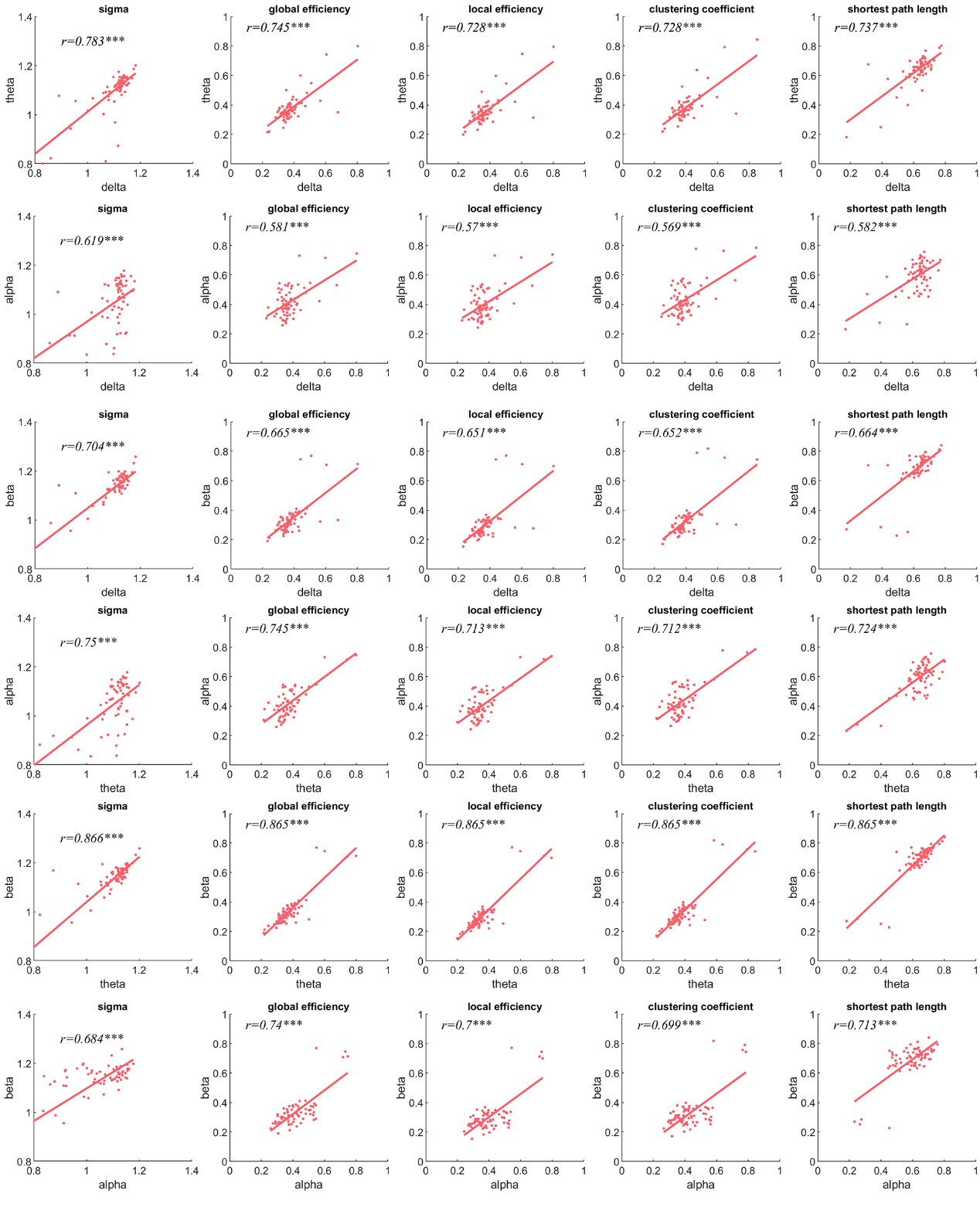


S.Fig.1 The correlations of graph parameters between different frequency bands in AIE group. *P<0.05, **P<0.01, P<0.001; r, Pearson’s correlation coefficient.


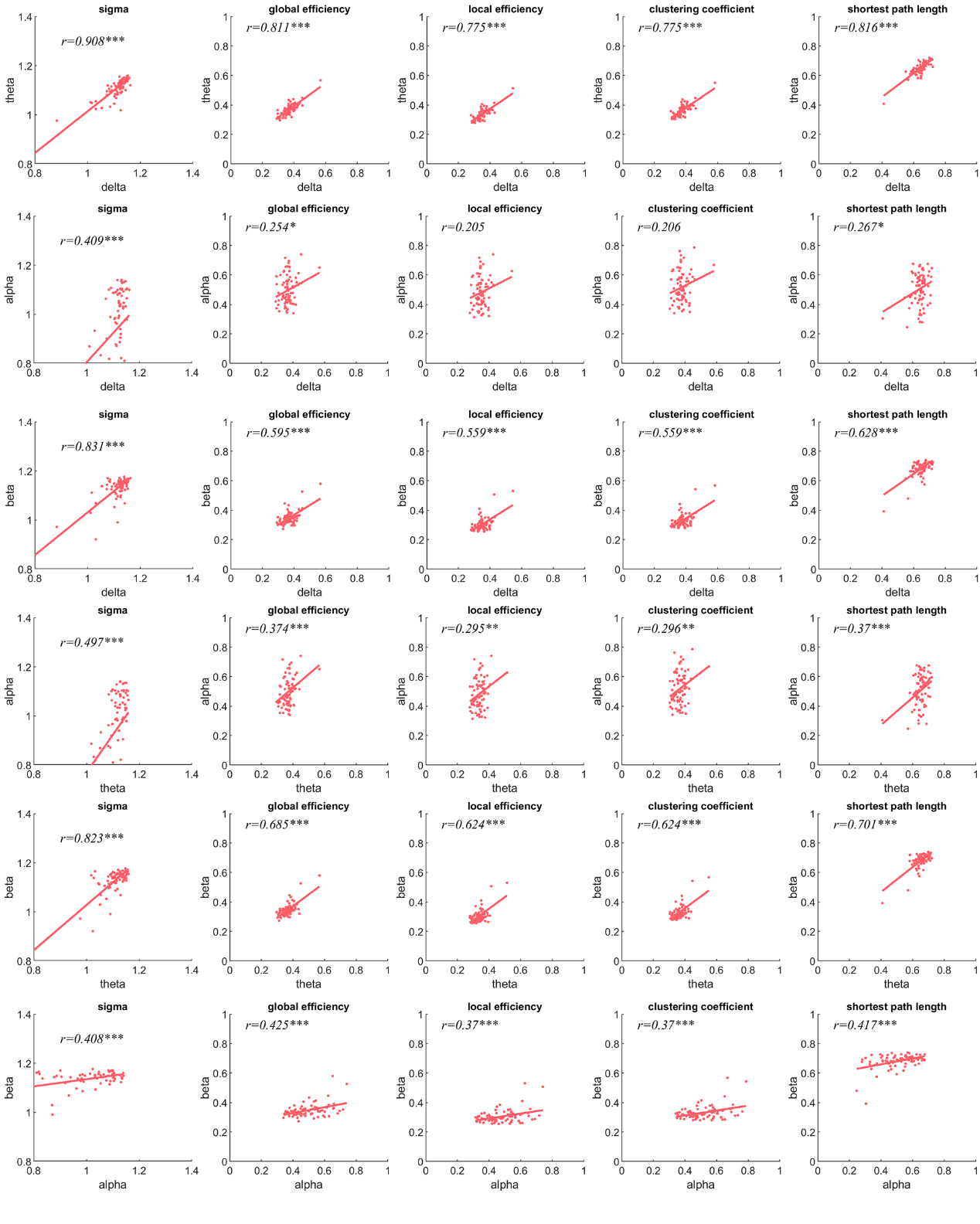


S.Fig.2 The correlations of graph parameters between different frequency bands in HC group. *P<0.05, **P<0.01, P<0.001; r, Pearson’s correlation coefficient.


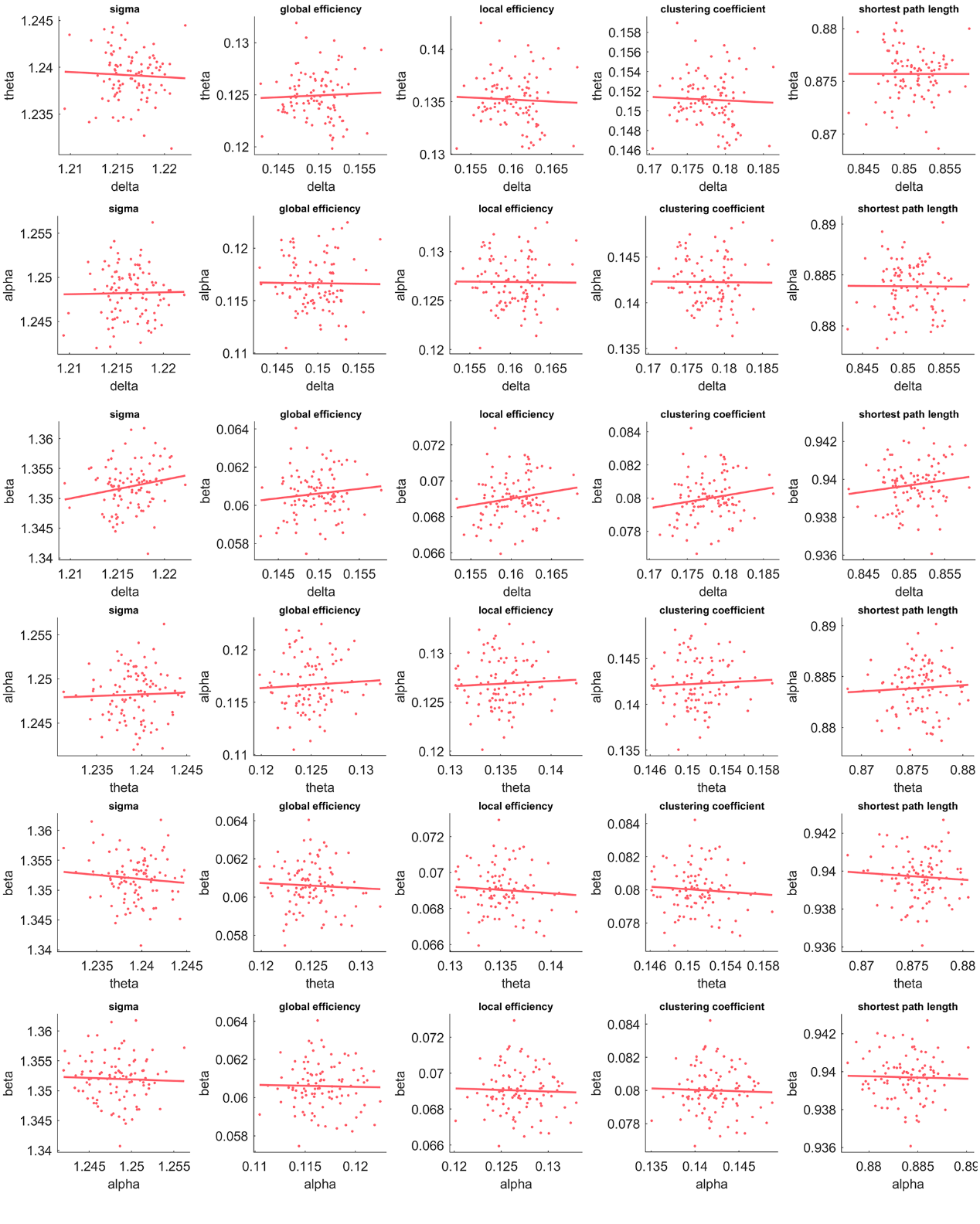


S.Fig.3 The correlations of graph parameters between different frequency bands in a group with simulated EEG data.


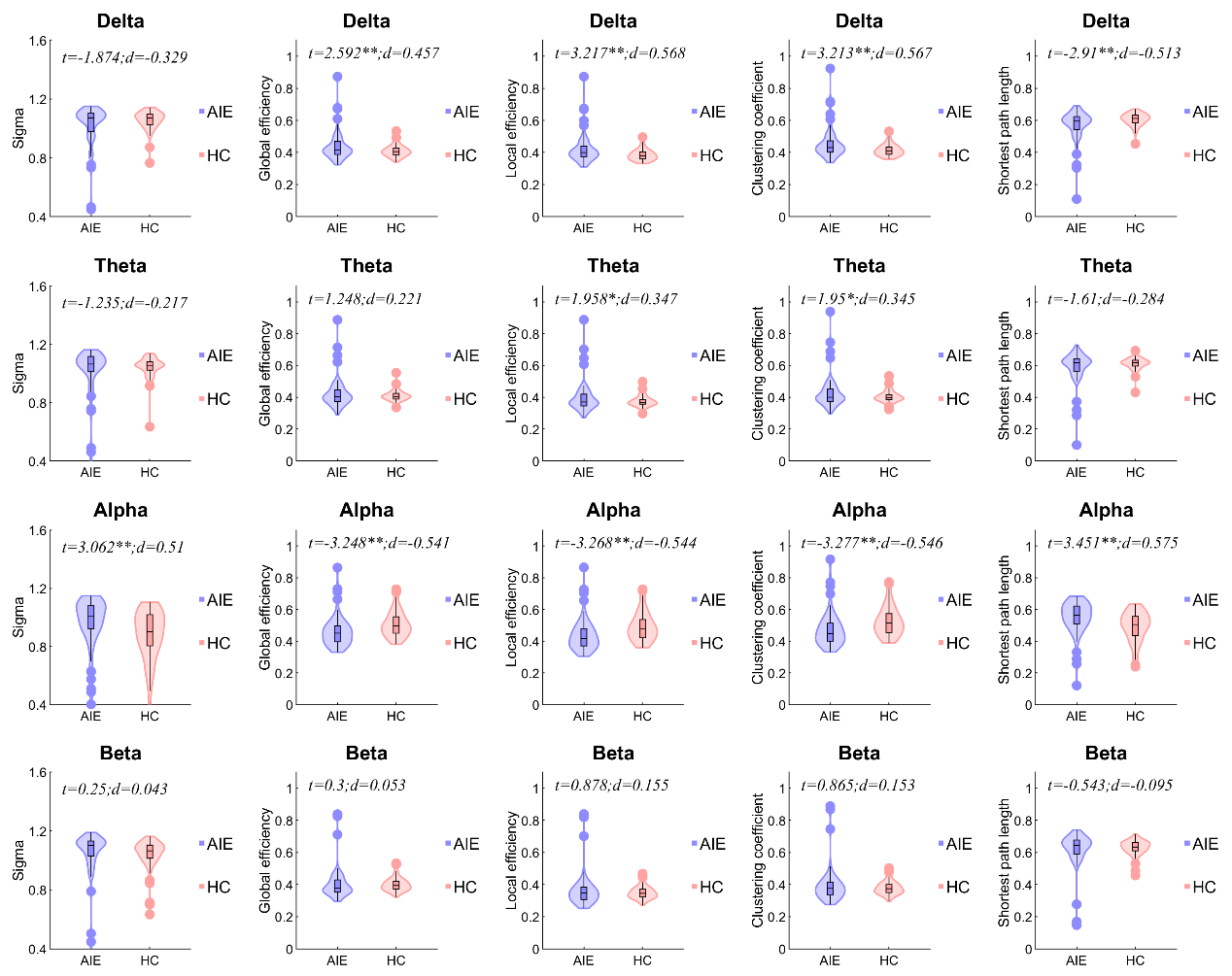


S.Fig.4 Comparisons of network properties between AIE group and HC group after surface Laplacian transformation of EEG data ^#^.

^#^Generalized linear models (GLM) with age and sex as covariates were used to assess AIE group and HC group differences. AIE, autoimmune encephalitis; HC, healthy control; *d*, the effect size Cohen’s *d*. **P*<0.05, ***P*<0.01, ****P*<0.001.
